# Influence of Urbanization on Body Size, Condition, and Physiology in an Urban Exploiter: A Multi-Component Approach

**DOI:** 10.1371/journal.pone.0135685

**Published:** 2015-08-13

**Authors:** Alizée Meillère, François Brischoux, Charline Parenteau, Frédéric Angelier

**Affiliations:** Centre d’Etudes Biologiques de Chizé (CEBC), UMR 7372 CNRS-Université de La Rochelle, Villiers-en-Bois, France; Università della Tuscia, ITALY

## Abstract

Consistent expanding urbanization dramatically transforms natural habitats and exposes organisms to novel environmental challenges, often leading to reduced species richness and diversity in cities. However, it remains unclear how individuals are affected by the urban environment and how they can or cannot adjust to the specific characteristics of urban life (e.g. food availability). In this study, we used an integrative multi-component approach to investigate the effects of urbanization on the nutritional status of house sparrows (*Passer domesticus*). We assessed several morphological and physiological indices of body condition in both juveniles (early post-fledging) and breeding adults from four sites with different levels of urbanization in France, Western Europe. We found that sparrows in more urbanized habitats have reduced body size and body mass compared to their rural conspecifics. However, we did not find any consistent differences in a number of complementary indices of condition (scaled mass index, muscle score, hematocrit, baseline and stress-induced corticosterone levels) between urban and rural birds, indicating that urban sparrows may not be suffering nutritional stress. Our results suggest that the urban environment is unlikely to energetically constrain adult sparrows, although other urban-related variables may constrain them. On the other hand, we found significant difference in juvenile fat scores, suggesting that food types provided to young sparrows differed highly between habitats. In addition to the observed smaller size of urban sparrows, these results suggest that the urban environment is inadequate to satisfy early-life sparrows’ nutritional requirements, growth, and development. The urban environment may therefore have life-long consequences for developing birds.

## Introduction

Pressures that human activities exert on the environment are steadily increasing and substantially affect ecosystem function [1]. In particular, consistent expanding urbanization irreversibly transforms the structure and ecological processes of natural habitats [2,3]. While some species seem to benefit from the urban environment (“urban exploiters”), many others seem unable to persist in cities (“urban avoiders”), and as a result, species richness and diversity overall is low in urban areas, especially for avian communities [3–6]. Indeed, urbanization exposes organisms to novel environmental challenges because of the specific characteristics of urban environments (e.g., resource availability, micro-climate, species interactions, disturbances, pollution; [7–13]). While the impact of urbanization on communities has been well documented [3–6,14,15], the mechanisms underlying organisms’ responses to urbanization are still poorly understood [8,16,17]. Yet, the modified environmental conditions of urban environments expose organisms to new selective pressures that are likely to affect wild vertebrates. For instance, studies have reported differences in behavior, morphology, and physiology between urban and non-urban populations (e.g., [18–23]) in a large range of species. Because the ability to successfully adapt to urban-related environmental changes can vary with species’ ecological and life history traits (e.g., dispersal ability, behavioral flexibility, diet, stress tolerance, annual fecundity [15,24,25]), organisms’ responses to urbanization differ highly among species. In a rapidly urbanizing world, it is crucial to understand not only how free-living organisms are affected by the urban environment, but also how they can or cannot adjust to its constraints.

Among other factors, food availability can be dramatically modified in cities [2,26,27]. Food types, quantity, and predictability certainly differ between rural and urban areas, and this is likely to have huge effect on individual fitness because energetic status is known to affect survival, reproductive performance, and the development of wild vertebrates [28–30]. However, the ultimate consequences of urban food resources on free-living organisms remain subject to debate [30–32]. First, food availability and predictability can be increased in cities compared to natural habitats because of human activities that consistently provide reliable food year-round [26]. Therefore, one could expect urban vertebrates to benefit from their environment and to be in overall good body condition (i.e., energetic state of an individual). Second, high food predictability could counter-intuitively have the opposite influence on body condition. Accumulating body reserves serves as a buffer against unpredictable temporary food shortage in birds [33,34], but is also associated with important metabolic and/or locomotory costs [35,36]. Therefore, individuals are predicted to reduce their body mass when living in a highly predictable environment where food shortages are scarce (i.e., adaptive mass regulation; reviewed in [37]), such as urban environments. Finally, although food quantity may not be impoverished in cities, high urban avian density could lead to strong inter-individual competition, and therefore, to poor food availability, overall [26,38]. In addition, urban wild vertebrates might rely on human-provided food (e.g., birdseed, refuse) because natural food is limited in terms of quantity and quality in cities [39–41]. Such food sources may be of insufficient quality and might not cover all nutritional requirements of urban wild vertebrates, especially during critical life-history stages (e.g., nestling or juvenile stages) or costly life-history events (e.g., reproduction) [27]. For these reasons, an urban diet may be insufficient to sustain energetic demands, therefore leading to poor body condition in urban individuals.

Because the same body condition pattern can result from different causes, assessing the exact energetic situation of urban wild vertebrates is challenging. To disentangle all these hypotheses, it is necessary to measure complementary indices of body condition. In addition to classical morphometric indices [42,43], further proxies for condition should be used, such as fat and pectoralis muscle scores [44–46]. These scores can aid in understanding whether differences in body condition are associated with energetic constraints or with an adaptive reduction of body mass [47,48]. Individuals are predicted to maintain their muscle mass and to preferentially reduce their fat stores when reducing their body mass for adaptive purposes, such as migration or reproduction [49,50]. However, both fat and muscle stores are predicted to decline when individuals become energetically constrained [49,51]. Because of the determinate growth of some vertebrates (e.g., birds, mammals), morphological measures are also crucial to consider when assessing the energetic situation of developing individuals [52]. Finally, other physiological indices can also be useful to understand the impact of specific environmental conditions on individual energetic status [16,22,53,54]. For instance, hematocrit is known to vary with energetic demands [55], and low hematocrit is also thought to be associated with survival costs in birds [56,57]. Therefore, low hematocrit could be an indicator of poor health status [55,58], and as a consequence, could provide a useful indicator of potential energetic constraints in urban birds. Likewise, corticosterone (hereafter CORT, the primary avian stress hormone) is an important mediator of allostasis, and CORT levels and the adrenocortical stress response are strongly related to the energetic status of an organism [59,60]. Thus, fasting usually triggers CORT secretion [61–65] whereas food intake is associated with a reduction of CORT levels [66,67]. Interestingly, the relationship between CORT levels and body condition is non-linear and CORT levels dramatically increase only when individuals reach a low condition threshold that is associated with nutritional stress [61,68]. Indeed, baseline (i.e., CORT levels in the absence of acute stressful events) and stress-induced CORT levels are known to increase when individuals are fed with food that does not allow sustaining their nutritional needs either quantitatively or qualitatively [69,70]. Moreover, elevated CORT levels are often associated with an increased risk of mortality [68,71–73]. Consequently, CORT levels can be a relevant addition to body condition when assessing the nutritional status of individuals [72,74].

To date, most studies have focused only on a limited number of body condition indices, a limited number of sites, a small sample size, and/or a single life-history stage, therefore making it difficult to fully assess the impact of urbanization on the nutritional status of wild vertebrates (but see [53]). Some studies have reported that urban individuals are in poorer condition than rural ones [75–80], but others have not found this difference [53,77]. The results regarding physiological indices of individual energetic condition are likewise inconsistent [16,19,23,53,81–90]. Because of these discrepancies, it remains difficult to determine whether urban birds are constrained by their environment or not. The house sparrow (*Passer domesticus*) is among the best examples of “urban exploiter” species, and is particularly well suited to investigate this question. Although certainly one of the most successful birds in the urban environment, the house sparrow has undergone population declines in the last several decades, particularly in European cities [91,92] and recent studies have suggested that poor food conditions may be one of the drivers of these declines [53,78,93]. Studies assessing the impact of urbanization on sparrows’ condition have shown that urban adult sparrows are generally in poorer condition than rural ones during the non-breeding season [77–79]. However, these results are not entirely supported by a more recent study [53], that reported no negative effect of urbanization in adult house sparrows. Similarly, studies investigating the influence of urbanization on CORT levels have also found conflicting patterns [53,84,94]. Furthermore, the constraints of urban life are likely to primarily affect individuals during critical life-history stages (e.g., early post-fledging period) and/or during costly stages of the annual cycle (e.g., reproduction), but to date, these periods have been overlooked. Because of the inconsistency of findings, it seems particularly important to investigate whether similar patterns are observed in other geographic locations, and also during constraining life-history stages.

In this study, we investigated the impact of urbanization on the nutritional status of free-living house sparrows using an integrative multi-component approach. We measured several morphological, hematological and hormonal indices of body condition in four populations of house sparrows (*Passer domesticus*), from sites with different levels of urbanization in France, Western Europe. We focused on both juveniles (i.e., young that have fledged during that season, before their first molt) and breeding adults to understand whether the impact of urbanization on wild birds is especially apparent during the post-fledging period (when the birds are inexperienced) and the reproductive period (when energetic demands are increased) [46]. By monitoring complementary and relevant characteristics of adults and juveniles from four sites, we will test whether house sparrows benefit from the urban environment (hypothesis 1), are constrained by the urban environment (hypothesis 2), adaptively reduced their body mass because of predictable urban food availability (hypothesis 3), and if the observed pattern differs between life-history stages. As explained earlier, each of these non-mutually exclusive hypotheses should be associated with specific patterns regarding our variables of interest (Table 1).

**Table 1:** Predicted response of morphological and physiological variables according to the different hypotheses of the impact of urbanization on the nutritional status of free-living house sparrows.

	Hypotheses		
Variable	Beneficial (Hyp. 1)	Constraining (Hyp.2)	Predictable (Hyp. 3)
**Morphological**			
Body size	+	-	Ø
Body condition	+	-	-
Fat score	+	-	-
Muscle score	+	-	Ø
**Physiological**			
Baseline CORT	-	+	Ø
Stress-induced CORT	-	+	Ø
Hematocrit	+	-	Ø

Symbols summarize the differences that should be found between urban and rural birds for each hypothesis (+ higher in urban relative to rural individuals, -lower in urban relative to rural individuals, Ø similar between urban and rural individuals).

## Materials and Methods

### Ethics statement

This study was carried out in accordance with all applicable institutional and/or national guidelines for the care and use of animals. All experimental procedures were approved by the “Comité d’Ethique en Expérimentation Animale Poitou-Charentes”, France (authorization number: CE2012-7). Permits for the capture, sampling and banding of house sparrows were issued by the “Centre de Recherches sur la Biologie des Populations d’Oiseaux” (permit numbers 15077 and 13794 delivered respectively to AM and FA). When sampling occurred on public land (2 sites: CEBC and La Rochelle, see below), permission was granted by the responsible authorities, i.e. the “Préfecture de la Charente-Maritime”, the “Préfecture des Deux-Sèvres”, and the “Centre d’Etudes Biologiques de Chizé”. Capture of sparrows on private property (2 sites: Niort and Villefollet, see below) was carried out with the land owners’ specific permission.

### Study species and sites

During the breeding season 2013, we captured 110 house sparrows (68 adults and 42 juveniles) with mist-nets at four sites in Western France (two urban and two rural sites, Table 2). The two urban sites are located within two medium-sized cities (La Rochelle, 46°08’52.8”N, 1°09’12.7”W, 75,000 inhabitants, and Niort, 46°18’46.4”N, 0°28’44.3”W, 58,000 inhabitants) whereas the two rural sites are situated in sparsely populated areas, surrounded by agricultural areas (in the village of Villefollet, 46°07’37.7”N, 0°16’04.4”W, 200 inhabitants) or by forests (at the Centre d’Etudes Biologiques de Chizé—hereafter CEBC, 46°08′50.5”N, 0°25′34.2”W, ~100 inhabitants). To quantify the degree of urbanization at each capture site, we followed the method developed by [78] for house sparrows. Briefly, we used digital aerial photographs (GoogleMaps) of 1 km x 1 km areas around each capture site that we divided into 100 cells. We extracted five habitat characteristics: mean building density score, number of cells with high building density, number of cells with road, mean vegetation density score, and number of cells with high vegetation density (Table 2). We then calculated an “urbanization score” for each site using the PC1 value from a principal component analysis on the five habitat variables (Table 2). The PC1 accounted for 91.2% of the total variance and correlated positively with artificial surfaces (building and roads) and negatively with vegetation cover.

**Table 2:** Habitat characteristics of the capture sites and sample sizes.

	Habitat Characteristics						Sample sizes	
Capture Site	Mean building density score	Number of cells with high building density	Number of cells with road	Mean vegetation density	Number of cells with high vegetation density	Urbanization score (PC1)	Adults	Juveniles
CEBC	0.11	1	23	1.98	98	-2.50	21	12
Villefollet	0.45	11	48	1.72	74	-1.21	21	9
Niort	1.18	24	97	0.82	11	1.61	18	10
La Rochelle	1.22	36	95	0.62	11	2.10	17	11

Sites are listed in increasing order of urbanization (PC1 values from a principal component analysis conducted on the five habitat variables).

### Bird sampling

Adult and juvenile sparrows were captured by “permanently monitored passive netting” as detailed in [95]. Sparrows were aged as adult or juvenile based on plumage characteristics [96]. Each capture site was sampled weekly: adults were captured from May 13 to August 11 and juveniles from June 16 to August 23. Capture dates did not differ between sites (ANOVA: adults: F_3,64_ = 2.147, p = 0.103; juveniles: F_3,38_ = 0.642, p = 0.593). The sample size for each site is summarized in Table 2.

In order to quantify baseline CORT concentrations, birds must be sampled within 3 min of capture [95,97]. We thus monitored the mist-nets permanently until a bird was captured. Immediately after capture, the bird was extracted from the mist-net and blood sampled as quickly as possible (mean ± SE: 2 min 39 sec ± 2 sec; range: 1 min 13 sec—3 min 45 sec). Blood samples were collected from the alar vein using a 27-gauge needle and heparanized microcapillary tubes (up to 150 μL for CORT assay and 10 μL for hematocrit measurement). CORT levels measured at the first blood sampling were not related to handling time (*r* = 0.008, p = 0.714, n = 110), and were therefore considered to be representative of “baseline CORT” levels. To measure the stress response, the bird was then kept in a cloth bag and a second blood sample was collected after 30 min (standard capture/restraint stress protocol; [98]). This second sample reflects the maximum CORT level (“stress-induced CORT”). Finally, the bird was banded with a numbered metal ring, weighed, measured (see below for details), and released at its site of capture.

### Morphological and hematocrit measurements

All birds were weighed (electronic balance: ± 0.1 g), and their wing length (steel rule: ± 1 mm) and tarsus lengths (caliper: ± 0.1 mm) were measured. For consistency and to avoid potential methodological bias, morphological and hematocrit measurements were all collected by AM. The repeatability of the morphological measurements was very high (intra-class correlation coefficient *R*: tarsus length: *R* = 0.963; wing length: *R* = 0.999; n = 17). Additionally, we recorded fat and muscle scores as detailed in [44–46]. To assess birds’ body condition, we used the “scaled mass index” (hereafter SMI) as recommended by [99,100]. The SMI adjust the mass of all individuals to that expected if they had the same body size [99]. We used tarsus length to calculate the SMI because it best correlated with body mass (tarsus length: *r* = 0.444, p < 0.001; wing length: *r* = 0.289, p < 0.001). The relationship between body mass and tarsus length was similar for males and females (Likelihood ratio = 1.751, p = 0.186) but different for adults and juveniles (Likelihood ratio = 9.039, p = 0.003), thus the SMI was calculated separately for adults and juveniles. The SMI was computed for each *i* individual as follows:

$${\text{SMI}}_{\text{i}}={\text{M}}_{\text{i}}\times {\left(\frac{{\text{L}}_{0}}{{\text{L}}_{\text{i}}}\right)}^{\text{b}}$$

where M_*i*_ and L_*i*_ are, respectively, the body mass and the tarsus length of the individual *i*, L_*0*_, the arithmetic mean value of tarsus length for the whole study population (adults: L_*0*_ = 18.76 mm, n = 68; juveniles: L_*0*_ = 18.11 mm, n = 42) and *b* the slope estimate of a standardized major axis (SMA) regression of log-transformed body mass on log-transformed tarsus length (adults: *b* = 1.35; juveniles: *b* = 2.20). Finally, we also measured hematocrit levels as an indicator of condition and health status [55,58]. Hematocrit was determined by centrifuging blood in a microcapillary tube (11000 rpm, 3min): the volume of red blood cells was expressed as a percentage of the total blood volume.

### Molecular sexing and hormone assay

Blood samples were centrifuged (4500 rpm, 7min), and plasma and red blood cells were separated and stored at -20°C until analyzed. Adult house sparrows were sexed visually (plumage characteristics; [96]), but the sex of juveniles was determined by molecular sexing as detailed in [101]. Plasma concentrations of CORT were measured in duplicate by radio-immunoassay, as previously described [102]. The minimum detectable CORT level was 0.83 ng.mL^-1^, and the intra- and inter-assay coefficients of variation were 7.07% and 9.99% respectively. All laboratory analyses were performed at the Centre d’Etudes Biologiques de Chizé (CEBC).

### Data analysis

All statistical analyses were performed in R 3.1.0 [103]. First, we fitted general linear models (GLMs; normal errors and identity link function) to test whether body size and condition differed between capture sites (response variables: tarsus length, wing length, body mass, SMI, fat score or muscle score). We used “site” (four-level factor from least to most urbanized: CEBC, Villefollet, Niort and La Rochelle) as explanatory variable, and included “age” (two-level factor: adult and juvenile; except for SMI where adults and juveniles were analyzed separately) and “sex” (two-level factor: male and female), as covariates. Each full model also included all 2-way interactions. For body mass and SMI analyses, we also included “time” of day as a covariate. Second, we fitted GLMs with normal errors and identity link function to determine whether physiological parameters differed between capture sites (response variable: baseline CORT, stress-induced CORT or hematocrit levels). Each full model included site, age, sex, and time of day (and a squared term: “time²” for the CORT models) and all 2-way interactions between site, age and sex. Baseline and stress-induced CORT levels were log_10_-transformed to ensure the normality of model residuals, but we present non-transformed values to facilitate interpretation. We did not include the “capture date” in the analyses because this variable differed highly between age classes and including age and capture date in the same models could lead to biased results (i.e. multicollinearity; [104]). Importantly, capture dates did not differ between sites (ANOVA, all p > 0.103, see above for details).

We performed all our model selection using a stepwise approach starting from the full model including all the independent variables and interactions. We used likelihood ratio test and removed non-significant factors and covariates one at a time until we reached the most parsimonious model. Tukey’s HSD (Honest Significant Difference) post-hoc tests were used to conduct pairwise comparisons between capture sites. All models were checked for assumptions of equal variances and normality of residuals.

## Results

### Body size and condition

**Fig 1:**
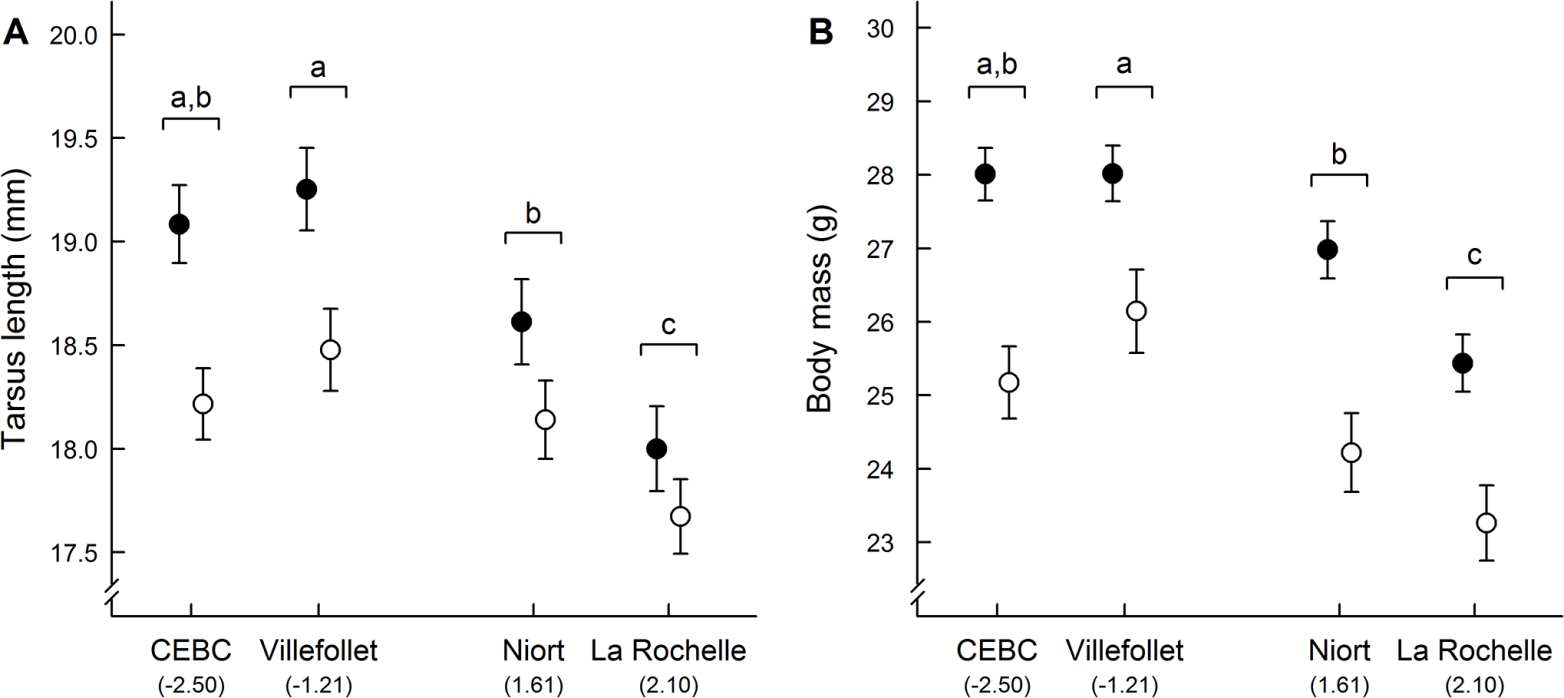
Mean ± SE (A) tarsus lengths and (B) body mass of sparrows captured in 4 sites with different levels of urbanization. Sites are ordered from least to most urbanized (PC1 values) with two rural (CEBC and Villefollet) and two urban sites. Filled circles represent adults and open circles represent juveniles (n = 110, see Table 2 for details). Differing letters indicate statistical difference between sites for both adults and juveniles (Tukey’s HSD test).

Both tarsus and wing lengths were significantly longer in adults than in juveniles (Table 3A and 3B, Fig 1A), and wing length was also significantly longer in males than in females, for adults only (Table 3B). Body mass also differed between age classes (Table 3C, Fig 1B), with adults being significantly heavier than juveniles. Wing length was not affected by the capture site (GLM: F_3,101_ = 0.860, p = 0.465), and the “site × sex” (F_3,97_ = 0.931, p = 0.429) or “site × age” (F_3,100_ = 2.140, p = 0.100) interactions. However, tarsus length and body mass significantly differed between sites (Table 3A and 3C), with sparrows from urban sites (Niort and La Rochelle) being generally smaller and lighter than rural ones (CEBC and Villefollet). Specifically, for all significant pairwise comparisons between sites, sparrows captured in more urbanized sites had smaller tarsus and weighed less than those captured in less urbanized sites (Fig 1A and 1B). Moreover, the “site × age” and “site × sex” interactions were not significant, suggesting that the influence of capture site on tarsus length and body mass did not significantly differ between adults and juveniles (tarsus: F_3,98_ = 0.746, p = 0.526; body mass: F_3,97_ = 0.756, p = 0.522) or males and females (tarsus: F_3,101_ = 0.908, p = 0.440; body mass: F_3,100_ = 1.464, p = 0.229).

**Table 3:** Minimum adequate models when investigating the influence of capture site on several morphological parameters.

Dependent variable	Independent variable	df	F	p-value
A) Tarsus length	Site	3,105	10.800	< 0.001
	Age	1,105	17.846	< 0.001
B) Wing length	Sex	1,106	51.086	< 0.001
	Age	1,106	69.511	< 0.001
	Sex × Age	1,106	8.887	0.004
C) Body mass	Site	3,105	15.133	< 0.001
	Age	1,105	59.771	< 0.001
D) Fat score	Site	3,102	1.2715	0.288
	Age	1,102	4.282	0.041
	Site × Age	3,102	2.816	0.043
E) Muscle score	Site	3,103	4.615	0.005
	Sex	1,103	13.321	< 0.001
	Age	1,103	0.425	0.516
	Sex × Age	1,103	8.150	0.005

Models were selected by using a stepwise approach starting from the full models (including site, age, sex, and interactions) and removing independent variables with P > 0.10.

**Fig 2:**
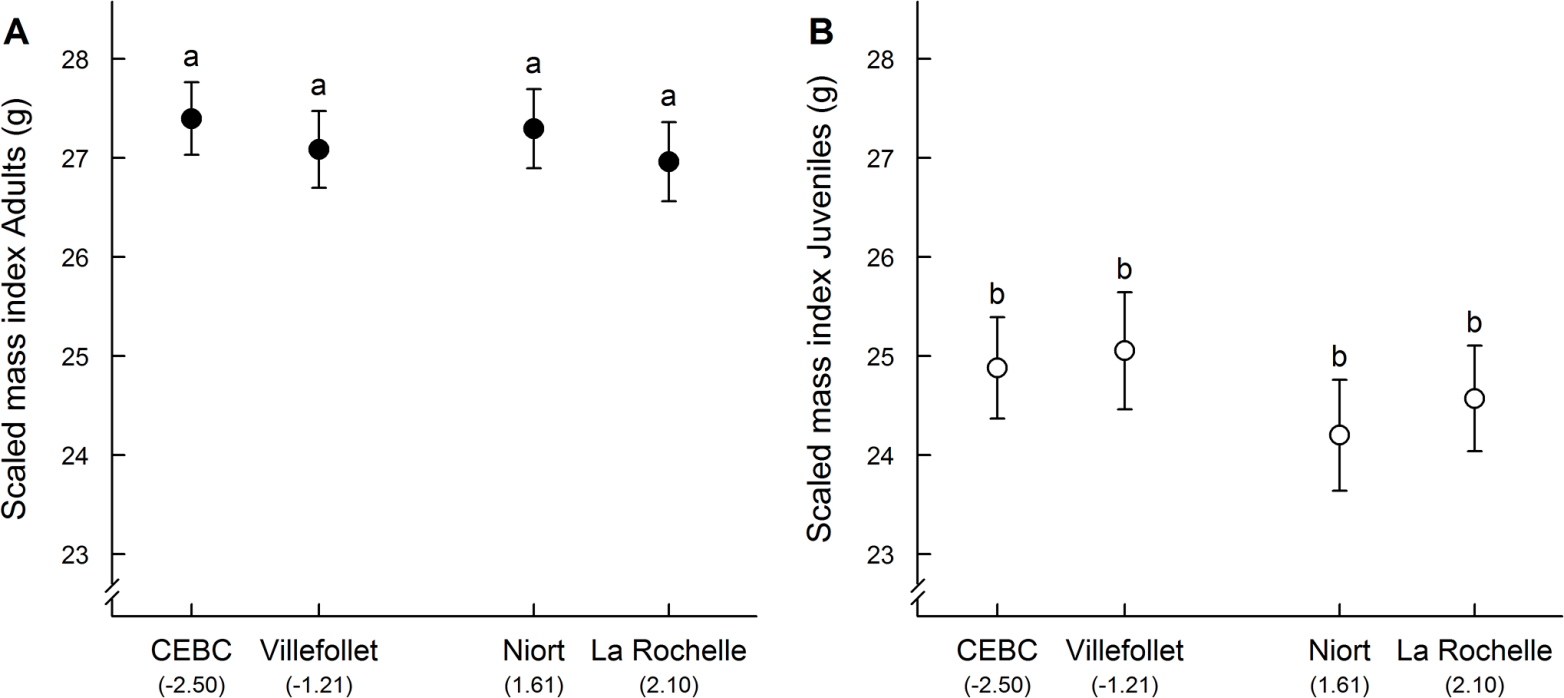
Mean ± SE scaled mass index values of (A) adult and (B) juvenile sparrows captured in 4 sites with different levels of urbanization. Sites are ordered from least to most urbanized (PC1 values) with two rural (CEBC and Villefollet) and two urban sites. Filled circles represent adults and open circles represent juveniles (n = 110, see Table 2 for details). The SMI did not significantly differ between sites as indicated by the similar letters (Tukey’s HSD test).

The SMI did not significantly differ between sites for both adults (GLM: F_3,63_ = 0.237, p = 0.870, Fig 2A) and juveniles (GLM: F_3,37_ = 0.624, p = 0.604, Fig 2B). Moreover, the SMI was not affected by sex or time of day (all p > 0.307 for both age classes).

**Fig 3:**
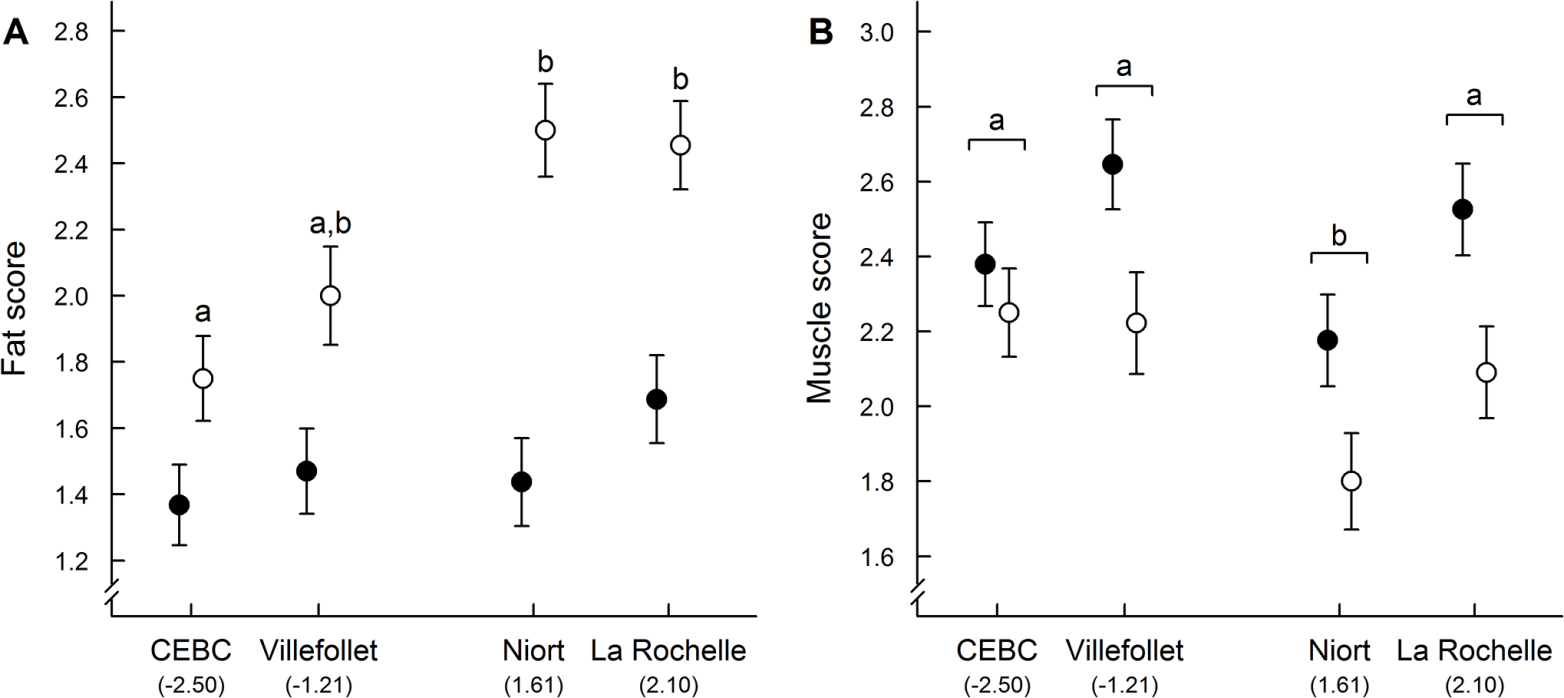
Mean ± SE (A) fat and (B) muscle scores of sparrows captured in 4 sites with different levels of urbanization. Sites are ordered from least to most urbanized (PC1 values) with two rural (CEBC and Villefollet) and two urban sites. Filled circles represent adults and open circles represent juveniles (n = 110, see Table 2 for details). Differing letters indicate statistical difference between sites for juveniles only (A) or for both adults and juveniles (B) (Tukey’s HSD test).

Adults had significantly lower fat score than juveniles (Table 3D) and higher muscle score than juveniles in males but not in females (Table 3E). There was an effect of the “site × age” interaction on fat score (Table 3D). Specifically, fat score significantly differed between sites in juveniles only, with sparrows captured in the less urbanized site (CEBC) having lower fat score than those captured in the two urban sites (Niort and La Rochelle, Fig 3A). Juveniles captured in Villefollet (rural) also had lower fat scores than those captured in Niort and La Rochelle (urban) but the differences were marginally significant (Tukey’s HSD test: Villefollet vs. Niort: p = 0.067; Villefollet vs. La Rochelle, p = 0.103). Finally, the site of capture had a significant effect on muscle score (Table 3E), but the observed differences were independent of urbanization: sparrows captured in Niort had lower muscle score than sparrows captured in the three other sites (Fig 3B).

### Physiological parameters

**Fig 4:**
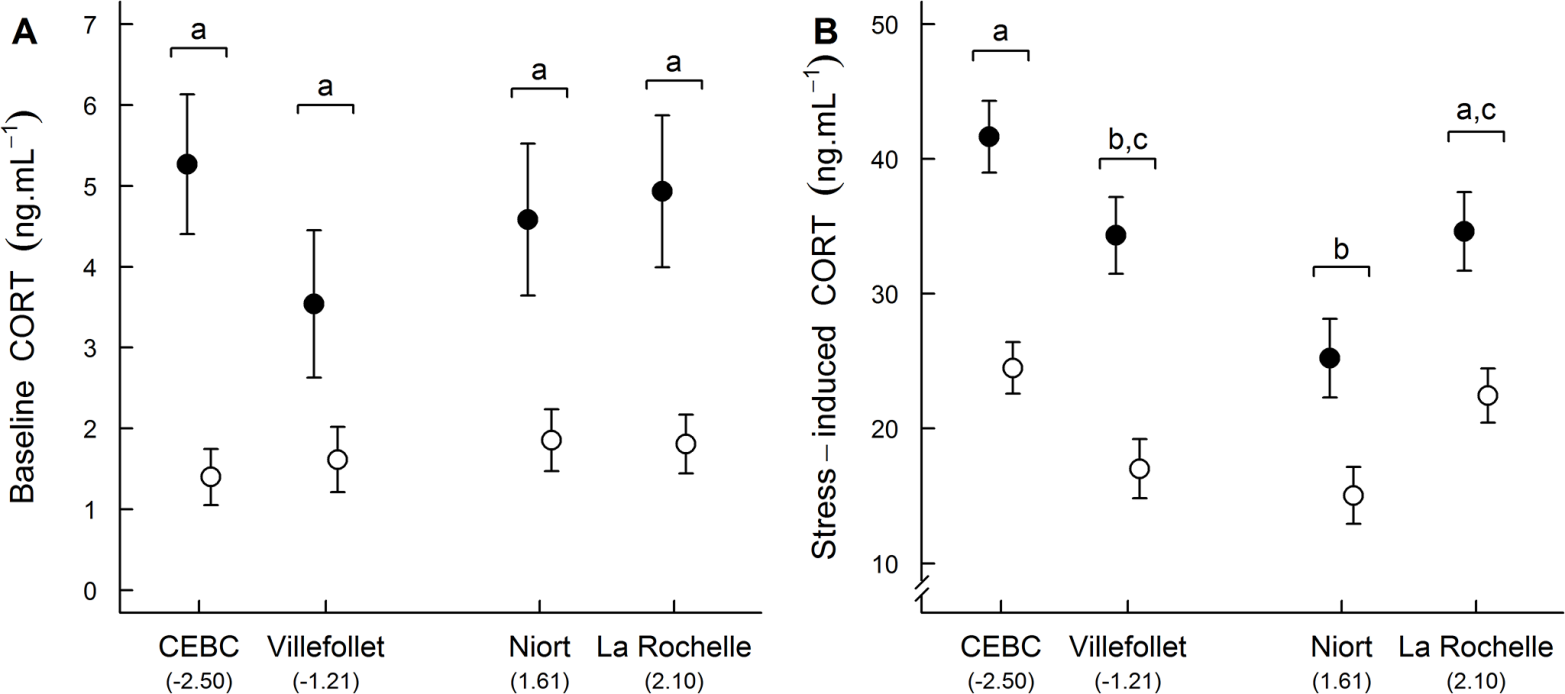
Mean ± SE (A) baseline and (B) stress-induced CORT levels of sparrows captured in 4 sites with different levels of urbanization. Sites are ordered from least to most urbanized (PC1 values) with two rural (CEBC and Villefollet) and two urban sites. Filled circles represent adults and open circles represent juveniles (n = 110, see Table 2 for details). Differing letters indicate statistical difference between sites for both adults and juveniles (Tukey’s HSD test).

Baseline CORT levels did not significantly differ between capture sites (F_3,103_ = 0.273, p = 0.845, Fig 4A) and were not affected by sex (F_1,102_ = 0.378, p = 0.540), and the “site × sex” (F_3,98_ = 0.293, p = 0.831) or “site × age” (F_3,95_ = 0.153, p = 0.927) interactions. However, baseline CORT levels were significantly lower in juveniles than in adults (Table 4A, Fig 4A).

**Table 4:** Minimum adequate models when investigating the influence of capture site on several physiological parameters.

Dependent variable	Independent variable	df	F	p-value
a) Baseline CORT	Age	1,106	29.482	< 0.001
	Time	1,106	5.193	0.025
	Time²	1,106	5.661	0.019
b) Stress-induced CORT	Site	3,104	9.393	< 0.001
	Sex	1,104	4.932	0.029
	Age	1,104	59.933	< 0.001
c) Hematocrit	Age	1,108	19.166	< 0.001

Models were selected by using a stepwise approach starting from the full models (including site, age, sex, time, time² and interactions) and removing independent variables with P > 0.10.

Site, age, and sex had a significant effect on stress-induced CORT levels (Table 4B). Stress-induced CORT levels were significantly lower in juveniles than in adults (Fig 4B), and lower in males than in females. Stress-induced CORT levels also significantly differed between capture sites (Table 4B), but the observed differences were not related to urbanization (Fig 4B). Specifically, stress induced CORT levels were significantly higher in one rural site (CEBC) than in the other rural site (Villefollet) and one urban site (Niort) and were significantly different between the two urban sites (lower in Niort than in La Rochelle; Fig 4B). Moreover, the “site × age” and “site × sex” interactions were not significant, suggesting that the influence of capture site on stress-induced CORT levels did not significantly differ between adults and juveniles (F_3,95_ = 0.440, p = 0.725) or males and females (F_3,98_ = 0.631, p = 0.597).

Hematocrit levels did not significantly differ between capture sites (F_3,104_ = 1.010, p = 0.391) and were not affected by sex (F_1,103_ = 0.008, p = 0.928) and the “site × sex” (F_3,100_ = 1.146, p = 0.800) or “site × age” (F_3,97_ = 0.461, p = 0.710) interactions. However, hematocrit levels significantly differed between age classes (Table 4C), with juveniles having a lower hematocrit than adults.

## Discussion

### Impact of urbanization on condition

In our study, we did not detect any evidence of an effect of urbanization on the SMI of both adults and juveniles, indicating that body condition of sparrows did not differ between urban and rural areas during these two critical stages (early post-fledging and breeding periods). Thus, despite the urban decline of some populations, the house sparrow does not seem to be particularly energetically constrained by the urban environment. Our results complement and are in accordance with another recent study that examined the influence of urbanization on the condition of house sparrows during other stages of the annual cycle in Hungary [53]. On the other hand, these results are not supported by other studies [78,79], which reported that urban sparrows are generally in poorer condition relative to their rural conspecifics. However, the conclusions of these studies were based on a condition index computed using mass-length residuals, a method that have recently been subject to debate [53,99,100]. In addition to the SMI, we also investigated complementary indices of body condition (i.e., fat and muscle scores, wing length, and several physiological indices) that all led to the same conclusion, strongly suggesting that urban sparrows are not suffering from nutritional stress. First, we did not find any consistent differences in fat and muscle scores between urban and rural adult sparrows, suggesting that urban adult sparrows were probably not energetically constrained [49,51]. Second, wing length did not differ between sites, thus, urban sparrows did not appear to face particularly important energetic constraints at the time of feather growth [105]. Third, we did not find any difference in hematocrit levels between urban and rural house sparrows, suggesting that urban sparrows did not suffer from anemia [55,58]. Finally, we did not detect any significant and clear effect of urbanization on baseline or stress-induced CORT levels, suggesting that urban sparrows are not energetically stressed [61–65,70]. Altogether, our results support the conclusion that urban sparrows are unlikely to be energetically constrained by the urban environment or to have adaptively reduced their body mass because of high food predictability in cities (rejecting hypothesis 2 and 3; Table 1).

Although we did not find any evidence suggesting that the urban nutritional environment affects house sparrows’ body condition, studies conducted on other species have reported that urban individuals are in poorer (e.g., Rufous-collared sparrow, *Zonotrichia capensis* [75]), similar (e.g., House Finch, *Haemorhous mexicanus* [87]) or better (e.g., Northern mockingbird, *Mimus polyglottos* [77]) condition compared to their rural conspecifics. These discrepancies highlight the contrasting abilities of different species to cope with the specific characteristics of urban environments. “Urban exploiter” species are expected to be in good body condition, as they might be better able to exploit urban food resources. However, in the present study, the house sparrow does not seem to benefit to a large extent from living in cities (rejecting hypothesis 1; Table 1), as urban sparrows were not in better condition relative to rural ones, and this idea is further supported by the recent decline of this species in European urban areas [91,92,106].

### Impact of urbanization on morphology

Although our study supports the idea that adult and juvenile house sparrows do not suffer from urbanization in term of body condition, we found large and significant differences in tarsus length along the urbanization gradient. Specifically, urban sparrows were smaller than their rural conspecifics and this finding is supported by other studies on house sparrow populations [53,78,79], and other bird species (e.g., [107]). However, a recent study investigating the effect of urbanization on avian morphology using paired urban and rural populations of European blackbirds (*Turdus merula*) along a latitudinal gradient, reported inconsistent (i.e., locality-dependent) difference between urban and rural populations [108]. In our study, sparrows captured in more urbanized sites had consistently smaller tarsi and weighed less than those captured in less urbanized sites. However, for one pairwise comparison there was a tendency for similar tarsus length between one urban and one rural site (NIORT vs. CEBC, see Fig 1), mostly because of the small size of juveniles captured at the CEBC. Here, it is important to acknowledge that our sample size was moderate for juveniles (42 juveniles), which could have limited our ability to detect an effect of urbanization and could explain the lack of difference between the two sites. However, this could also suggest that despite a strong effect of urbanization on sparrows’ morphology, this effect can be reduced or accentuated because of local specific characteristics of each site. Our results also highlight the importance of investigating more than one urban and one rural population to fully assess the influence of urbanization on wild vertebrates.

Interestingly, tarsus lengths differed among our studied populations, while wing lengths were similar. In house sparrows, the tarsus is almost fully grown at fledging, whereas wing length is mainly determined by the length of primary feathers that are replaced every year (post-nuptial molt) and a few weeks after fledging (the post-fledging molt) [96]. Thus, the smaller tarsi of urban birds probably results from energetic constraints on developing nestlings or fledglings. Several hypotheses could explain the production of smaller individuals in cities. First, it may arise from parents’ reproductive decisions (e.g., trade-offs between clutch size and nestlings’ body condition; [26]), but this seems unlikely in house sparrows because clutch sizes (a proxy for parental investment) have been shown to be similar in urban and rural habitats [109]. Second, the urban environment could constrain nestling development through, for example, a direct effect of low quality, low quantity, or contaminated food on growth [93,110], but also through a potential indirect effect of urban noise on the parental provisioning of the brood [111]. Finally, the smaller body size of urban birds may also result from adaptive divergences between urban and rural populations. For instance, differences in predator regime (e.g., strong predation in cities; [78,112]), micro-climate (e.g., urban heat island effect and Bergman’s rule; [108,113]) and/or migration tendencies (e.g., reduced migratory tendencies in cities and Seebohm’s rule; [108,114]) may favor individuals of smaller body size in urban environments. However, most of these hypotheses seem unlikely in house sparrows as we did not find evidence for smaller wing length in urban individuals, and further investigations would be needed to assess differences in predation pressure between urban and rural sites.

In addition to the smaller size of urban sparrows, we found significant differences in juvenile fat scores between capture sites, with urban juveniles having, surprisingly, higher fat scores than rural ones. These results could suggest that food types provided to nestlings and juveniles highly differed between urban and rural habitats. The difference in body reserves between urban and rural juveniles could thus indicate that urban sparrows might not feed their nestlings and young fledglings with arthropod prey only (i.e., the main component of nestling sparrows’ diet; [96]) because this type of food is less available in the urban environment [115]. Instead, they may incorporate fatter and highly accessible human-provided food in their diets [39,93,116]. Because such food could be inadequate or of insufficient quality (e.g., lower protein or calcium content) to satisfy all nestlings’ nutritional requirements, this may have negatively affected their growth and development [27,39,116]. Accordingly, Seress et al. (2012) reported that suburban house sparrows produce smaller fledglings than their rural conspecific in Central Europe [93]. Moreover, the results from their common garden and cross-fostering experiments support the idea that these differences result from different nestling diets in urban and rural habitats [93]. Altogether, our results and these studies suggest that urban house sparrows are constrained by their environment during their developmental phase at a large European scale (supporting hypothesis 2 during development only, Table 1).

Urban environments differ highly from natural habitats [3,8], and most of urban-associated factors are likely to affect several traits, such as morphology, in wild vertebrates. For instance, habitat structure, pollution levels, and climate can influence urban arthropod communities [115,117], as well as, human presence contributes to anthropogenic provision of food resources in cities [39–41]. As a result, divergences in diets, and associated developmental and morphological consequences, are likely to arise as a result of urbanization. Because conditions experienced during development influence phenotypic development and can shape individual life histories [52,118], the smaller body size in urban populations may have important consequences for fitness, and ultimately for populations viability. A smaller body size is often related to poorer developmental conditions and to poorer performance (survival and reproductive success) later in life [52,107,119,120]. The concomitant smaller size and decline of urban house sparrow populations in Europe supports this hypothesis. However, a smaller body size can also be adaptive under some circumstances if it allows an individual to better perform in its environment (reviewed in [121]). Since contradictory hypotheses can explain the smaller size of urban individuals, further investigations are needed to better assess whether smaller structural size in urban populations might be adaptive or might lead to important fitness costs.

### Impact of urbanization on stress physiology

We did not detect any evidence of an effect of urbanization on stress physiology, suggesting therefore that urban sparrows do not experience intense nutritional stress relative to rural sparrows. However, we found significant and large differences in the stress response between sites independently of the degree of urbanization, suggesting that the relationship between stress physiology and urbanization is complex and inconsistent. Accordingly, to date, no general and clear patterns have been revealed regarding the stress physiology of urban birds (reviewed in [16]). Studies have reported positive, negative and mainly null relationships between urbanization and stress-induced CORT levels (e.g., see [19,23,53,82,84,88,90]) and the observed effects not only differed among but also within species with differences depending on the sex, the life-history stage, or the year [16].

In addition to the energetic status, numerous intrinsic (e.g., sex, age, quality, reproductive status, species life history; [23,81,122–124]) and/or extrinsic factors (e.g., weather, predation risk, noise, pollution; [125–128]) can influence the CORT stress response and could have masked any general effect of urbanization on stress physiology [129]. Importantly, we found similar patterns of CORT secretion between sites for both breeding adults and juveniles indicating that breeding effort was not a confounding factor in our study [123]. Other studies have investigated the effects of urbanization on other measures of physiological stress (e.g., oxidative stress), and have also reported conflicting patterns (e.g. see [86,87]). Altogether, these studies highlight the importance of investigating multiple physiological components (e.g., oxidative stress, CORT stress response, parasite infection, carotenoid levels; [19,22,23,53,86,87,89,130]. Overall, the relationship between urbanization and avian stress physiology appears complex and the observed differences between sites are likely due to site-specific characteristics (e.g., parasites, pollution, predation) that may outweigh a potential effect of urbanization on these physiological components.

## Conclusions

We found no evidence of an effect of urbanization on the condition of house sparrows during two energy-demanding stages (early post-fledging and breeding periods). By using an integrative multi-component approach, incorporating morphological, structural and physiological measures from both breeding adults and juveniles from four different sites, we also demonstrated that adult urban sparrows are not energetically constrained by the urban environment. Conversely, our results strongly suggest that the urban nutritional environment is inadequate or of insufficient quality to satisfy all nutritional requirements of developing sparrows, and therefore, that the urban environments are likely to energetically constrain free-living birds during their developmental phase only. However, the long-term consequences of growing in an urban environment remain unclear and future studies should explore these questions by comparing urban and rural populations [22,54]. More generally, the study of the consequences of urban life on wildlife may benefit from adopting experimental approaches (e.g., see [19,93,111,116,126]) to disentangle the relative importance of confounding factors (e.g., food quality versus predictability and/or quantity, urban noise, pollution, predation) on performances. These experimental studies will also help ecologists to assess the mechanisms underlying organismal responses to urbanization. Finally, they will also provide unequivocal evidence to explain the recent population trends—positive or negative—of some urban vertebrates.

## Supporting Information

S1 Dataset Supporting data. (PDF)
