# Supplementary material for: Influence of Urbanization on Body Size, Condition, and Physiology in an Urban Exploiter: A Multi-Component Approach
Source: PLoS One. 2015 Aug 13;10(8):e0135685. doi: 10.1371/journal.pone.0135685 (PMC4535910; doi:10.1371/journal.pone.0135685)
Supplement: S1 Dataset — (PDF) [file pone.0135685.s001.pdf]

Supporting Data

Site	Date	DayTime	Sex	Age	Tarsus	Wing	Weight	Fat	Muscle	CortA	TimeA	CortB	Hemato
LaRochelle	04/07/2013	11:20	M	Ad	18.9	77.0	25.1	1	3	4.74	3.00	31.32	0.503
LaRochelle	18/07/2013	12:03	F	Ad	18.1	77.0	28.4	2	3	3.90	2.80	25.57	0.505
LaRochelle	18/07/2013	10:30	F	Ad	18.2	72.0	24.4	2	3	0.83	2.23	27.87	0.465
LaRochelle	04/07/2013	10:30	M	Ad	18.5	78.0	26.9	2	3	15.12	3.00	41.29	0.547
LaRochelle	04/07/2013	12:10	M	Ad	17.4	75.0	24.0	1	3	4.49	3.00	49.40	0.514
LaRochelle	19/06/2013	11:25	F	Ad	18.8	73.0	25.8	2	3	5.30	1.83	44.82	0.560
LaRochelle	04/07/2013	12:10	F	Ad	17.5	74.0	25.9	2	3	14.85	3.00	41.69	0.477
LaRochelle	19/06/2013	10:35	M	Ad	16.4	77.0	25.4	1	3	1.08	2.33	43.11	0.465
LaRochelle	04/07/2013	10:30	F	Ad	17.8	73.0	26.5	1	2	11.16	2.90	53.04	0.452
LaRochelle	06/08/2013	12:35	F	Ad	19.3	77.0	24.2	3	2	0.83	2.83	25.79	0.367
LaRochelle	31/07/2013	10:45	F	Ad	17.9	74.0	24.5	2	2	2.16	2.27	35.28	0.536
LaRochelle	31/07/2013	11:35	M	Ad	18.6	79.0	26.4	2	2	2.22	2.33	18.06	0.439
LaRochelle	31/07/2013	13:40	M	Ad	17.0	79.0	24.9	2	3	0.86	2.80	14.55	0.286
LaRochelle	19/06/2013	11:40	M	Ad	18.0	77.0	26.7	1	2	5.33	2.00	44.73	0.510
LaRochelle	19/06/2013	11:35	F	Ad	18.2	75.0	24.1	1	1	2.70	3.00	31.67	0.388
LaRochelle	06/08/2013	11:55	F	Ad	17.4	75.0	23.8	2	2	3.36	3.00	29.46	0.456
LaRochelle	06/08/2013	11:30	F	Juv	18.8	67.0	25.9	2	3	1.13	2.37	19.58	0.429
LaRochelle	06/08/2013	13:05	F	Juv	18.2	71.0	22.4	2	2	2.93	2.50	21.34	0.444
LaRochelle	06/08/2013	09:20	F	Juv	17.7	70.0	24.3	2	2	0.83	2.67	24.80	0.462
LaRochelle	18/07/2013	14:02	M	Juv	16.6	70.0	20.3	2	2	4.43	3.00	46.18	0.450
LaRochelle	06/08/2013	11:55	M	Juv	17.9	72.5	21.9	2	2	1.61	3.00	15.20	0.421
LaRochelle	06/08/2013	14:38	M	Juv	19.0	74.0	26.1	3	2	0.83	2.42	16.15	0.404
LaRochelle	14/08/2013	10:43	F	Juv	17.4	70.0	22.3	3	2	0.83	2.70	22.76	0.456
LaRochelle	06/08/2013	14:25	M	Juv	17.9	72.0	25.6	3	2	0.83	2.77	10.26	0.448
LaRochelle	21/08/2013	11:31	F	Juv	16.2	68.0	21.0	3	2	4.70	2.23	30.09	0.403
LaRochelle	23/08/2013	13:50	F	Juv	17.4	70.0	22.8	3	2	0.93	2.23	23.19	0.407
LaRochelle	23/08/2013	13:54	F	Juv	17.3	67.5	23.3	2	2	0.83	2.50	21.80	0.457
Villefollet	26/05/2013	19:22	F	Ad	20.8	71.0	29.3	1	3	5.97	3.00	61.43	0.516
Villefollet	14/07/2013	17:55	M	Ad	17.6	74.0	25.1	1	2	13.49	2.38	46.36	0.525
Villefollet	26/05/2013	20:15	F	Ad	18.9	76.0	30.3	2	3	1.05	3.00	41.27	0.624
Villefollet	11/06/2013	17:40	M	Ad	20.2	79.0	27.6	2	3	4.89	3.00	40.99	0.550
Villefollet	11/06/2013	17:05	M	Ad	20.7	78.0	30.8	2	3	0.83	2.42	38.30	0.514
Villefollet	02/06/2013	18:20	M	Ad	19.5	77.5	29.0	2	3	6.76	3.00	38.60	0.529
Villefollet	26/05/2013	19:20	M	Ad	19.3	76.0	26.8	1	3	5.36	2.80	45.59	0.565
Villefollet	16/06/2013	16:30	F	Ad	19.5	77.0	28.8	1	2	0.91	2.33	28.98	0.468
Villefollet	10/08/2013	20:10	M	Ad	19.4	78.0	30.9	2	3	1.91	3.00	15.81	0.435
Villefollet	11/08/2013	20:00	M	Ad	18.6	76.0	29.1	2	3	2.78	2.67	24.87	0.437
Villefollet	07/07/2013	19:35	M	Ad	19.9	80.0	27.7	2	3	3.74	2.25	33.66	0.442
Villefollet	25/07/2013	08:53	M	Ad	18.1	78.0	28.7	2	3	2.57	3.00	20.97	0.456
Villefollet	16/06/2013	18:25	M	Ad	19.3	79.0	28.3	1	3	2.04	2.95	40.06	0.488
Villefollet	05/08/2013	09:40	F	Ad	19.8	74.0	28.4	1	2	1.03	3.00	16.42	0.395
Villefollet	16/06/2013	17:40	M	Ad	18.0	74.0	25.0	1	3	1.68	3.00	29.83	0.537
Villefollet	05/08/2013	08:32	F	Ad	18.0	72.5	24.8	1	2	3.69	3.00	23.13	0.464
Villefollet	05/08/2013	09:35	F	Ad	19.7	74.0	25.7	1	2	1.48	3.00	28.19	0.313
Villefollet	12/08/2013	11:10	F	Juv	18.8	70.0	29.8	2	2	0.83	1.67	13.80	0.400
Villefollet	19/08/2013	09:43	F	Juv	17.9	66.0	24.5	2	3	1.00	2.93	20.20	0.484
Villefollet	19/08/2013	10:16	M	Juv	18.4	72.0	23.3	2	2	2.21	1.95	21.91	0.373

Villefollet	16/06/2013	18:25	M	Juv	18.6	76.0	26.0	2	3	0.90	2.68	16.31	0.447
Villefollet	18/08/2013	19:01	F	Juv	18.3	71.5	29.6	2	2	2.16	1.42	15.21	0.468
Villefollet	07/07/2013	17:15	F	Juv	18.1	71.0	26.2	2	2	1.87	2.95	18.06	0.472
Villefollet	21/07/2013	19:25	F	Juv	17.9	74.0	24.9	2	2	3.92	2.90	23.91	0.435
Villefollet	18/08/2013	19:30	M	Juv	19.1	70.0	25.2	2	2	0.83	1.67	18.69	0.421
Villefollet	18/08/2013	19:13	M	Juv	19.2	74.0	25.8	2	2	0.83	2.67	6.57	0.458
Niort	02/08/2013	10:10	F	Ad	18.1	74.0	26.0	2	2	1.76	2.57	27.38	0.445
Niort	02/08/2013	12:06	F	Ad	18.6	75.0	26.0	2	1	9.83	2.00	28.16	0.476
Niort	06/06/2013	15:50	M	Ad	19.8	78.0	27.9	1	2	6.37	2.53	23.45	0.528
Niort	06/06/2013	09:40	M	Ad	18.9	78.0	27.1	1	2	4.30	2.55	23.95	0.598
Niort	09/08/2013	13:53	F	Ad	17.5	73.0	26.6	2	2	9.09	1.22	19.77	0.420
Niort	23/05/2013	12:27	M	Ad	19.4	75.0	27.5	1	2	0.83	2.00	22.66	0.523
Niort	23/05/2013	15:17	F	Ad	19.2	76.0	28.3	1	2	0.83	1.78	15.11	0.517
Niort	02/08/2013	11:30	F	Ad	18.6	76.0	26.4	1	2	3.88	2.92	30.22	0.500
Niort	02/08/2013	12:04	M	Ad	19.6	80.0	27.4	1	2	10.70	2.50	33.27	0.471
Niort	06/06/2013	09:25	F	Ad	17.7	76.0	26.1	1	2	0.83	1.82	25.91	0.550
Niort	20/06/2013	13:34	M	Ad	18.6	76.0	26.9	1	3	0.83	2.83	20.09	0.471
Niort	23/05/2013	10:45	F	Ad	19.4	73.0	28.9	1	2	5.36	3.00	35.69	0.504
Niort	23/05/2013	11:15	F	Ad	18.7	74.0	29.7	2	3	6.89	3.00	34.77	0.493
Niort	02/08/2013	10:32	M	Ad	18.4	78.0	26.7	2	3	0.95	2.83	10.63	0.457
Niort	09/08/2013	09:50	F	Ad	18.0	75.0	24.6	2	2	3.75	3.00	34.73	0.553
Niort	07/08/2013	13:48	F	Ad	17.3	72.0	25.6	2	2	7.13	3.00	24.94	0.461
Niort	02/08/2013	12:15	M	Juv	18.7	73.0	25.6	3	2	5.37	1.33	18.44	0.455
Niort	01/08/2013	14:50	F	Juv	18.8	72.0	24.0	3	2	1.54	3.00	9.80	0.434
Niort	13/08/2013	11:10	F	Juv	18.7	71.0	21.7	2	1	1.93	3.00	25.11	0.295
Niort	29/07/2013	12:20	M	Juv	18.0	71.0	26.0	3	2	0.83	3.00	10.67	0.462
Niort	29/07/2013	12:20	M	Juv	17.6	71.0	22.9	3	1	0.83	3.75	12.95	0.502
Niort	29/07/2013	16:10	F	Juv	18.2	72.0	23.2	3	2	3.00	1.92	17.57	0.426
Niort	09/08/2013	11:34	F	Juv	17.5	71.0	24.4	2	2	1.53	1.92	15.95	0.480
Niort	13/08/2013	11:30	F	Juv	18.4	73.0	24.9	2	2	0.83	2.78	18.10	0.381
Niort	16/08/2013	12:15	F	Juv	17.9	72.0	25.6	2	2	1.85	3.00	16.14	0.438
Niort	05/07/2013	16:58	F	Juv	17.6	71.0	23.9	2	2	0.83	2.75	11.55	0.394
CEBC	24/07/2013	18:50	M	Ad	19.2	77.0	26.8	1	2	0.83	2.57	13.07	0.362
CEBC	24/07/2013	14:25	F	Ad	18.2	71.0	25.6	2	2	5.36	3.00	44.82	0.526
CEBC	21/05/2013	15:20	M	Ad	19.5	79.0	28.4	2	2	4.97	2.50	44.90	0.498
CEBC	13/05/2013	15:40	M	Ad	20.0	80.0	26.8	1	2	11.81	2.83	47.52	0.384
CEBC	23/07/2013	14:30	M	Ad	18.9	78.0	26.3	1	3	14.76	2.00	58.84	0.456
CEBC	22/07/2013	17:43	M	Ad	17.9	77.0	27.6	1	2	1.71	3.00	21.03	0.471
CEBC	21/05/2013	15:40	F	Ad	19.7	76.0	28.2	1	2	4.31	3.00	69.76	0.483
CEBC	15/05/2013	14:35	F	Ad	17.9	76.0	26.2	2	3	5.17	2.87	56.40	0.449
CEBC	06/06/2013	15:30	F	Ad	19.0	76.0	28.4	1	2	4.84	2.83	41.81	0.544
CEBC	21/05/2013	15:45	F	Ad	19.8	77.0	28.5	1	2	8.02	3.00	28.86	0.403
CEBC	04/06/2013	16:20	M	Ad	19.4	76.0	27.5	1	3	8.17	3.08	37.38	0.527
CEBC	27/07/2013	15:50	M	Ad	17.2	78.0	26.6	2	3	4.33	2.92	21.77	0.500
CEBC	24/05/2013	14:56	M	Ad	19.9	80.0	31.5	1	3	3.98	2.43	47.80	0.495
CEBC	25/06/2013	15:45	F	Ad	19.5	76.0	30.6	2	2	6.16	2.88	40.63	0.500
CEBC	27/06/2013	16:35	F	Ad	20.0	74.0	30.2	2	2	2.89	3.00	36.63	0.463
CEBC	04/06/2013	15:10	F	Ad	19.4	74.0	27.5	1	2	1.34	2.27	39.39	0.475
CEBC	12/06/2013	09:40	F	Ad	19.4	75.0	29.3	1	3	2.69	2.00	63.94	0.500
CEBC	23/07/2013	19:15	M	Ad	19.5	77.0	28.6	2	3	1.37	3.00	42.22	0.461
CEBC	06/06/2013	11:25	F	Ad	18.2	72.5	27.6	1	2	7.37	2.80	36.38	0.449
CEBC	11/07/2013	15:50	F	Juv	17.5	71.0	22.8	1	2	1.31	3.00	33.66	0.436
CEBC	22/07/2013	18:34	M	Juv	18.0	71.0	26.1	2	3	0.83	3.00	16.87	0.518

CEBC	19/07/2013	15:24	F	Juv	18.4	70.0	25.7	1	3	3.27	2.78	17.48	0.313
CEBC	22/07/2013	18:59	M	Juv	18.2	71.0	23.9	2	2	0.83	2.50	21.45	0.428
CEBC	13/08/2013	20:22	M	Juv	18.8	72.0	26.0	2	2	2.22	3.00	28.73	0.427
CEBC	13/08/2013	19:23	F	Juv	18.5	72.0	26.7	2	2	1.23	3.00	25.24	0.448
CEBC	15/08/2013	19:56	M	Juv	18.0	70.0	24.2	1	2	0.83	2.08	22.37	0.406
CEBC	15/08/2013	20:16	M	Juv	17.6	67.0	25.1	2	2	1.59	2.33	29.64	0.443
CEBC	05/08/2013	18:50	F	Juv	19.2	71.0	25.6	2	2	1.78	3.00	29.13	0.500
CEBC	05/08/2013	18:50	F	Juv	18.1	71.0	26.3	2	3	1.12	3.00	30.91	0.455
CEBC	22/07/2013	19:11	M	Juv	18.2	70.0	24.2	2	2	0.83	3.00	17.36	0.254
CEBC	13/08/2013	20:33	M	Juv	18.1	71.0	25.5	2	2	0.96	3.00	18.12	0.467
